# Rapid, sensitive, and highly specific detection of *Mycoplasma pneumoniae* and its mutation using the RPA-CRISPR/Cas13a system

**DOI:** 10.1128/spectrum.01793-25

**Published:** 2026-03-31

**Authors:** Yanli Ren, Meng Hong, Guodi Wu, Shanshan Wu, Lin Chen, Jing Wang, Haihong Zhu, Zhaoyang Peng, Zhi Chen

**Affiliations:** 1 State Key Laboratory for Diagnosis and Treatment of Infectious Diseases, National Clinical Research Center for Infectious Diseases, National Medical Center for Infectious Diseases, Collaborative lnnovation Center for Diagnosis and Treatment of lnfectious Diseases, The First Affiliated Hospital, Zhejiang University School of Medicine 71069, Hangzhou, China; 2 Yuhang Institute of Medical Science Innovation and Transformation, Hangzhou, China; 3 Department of Clinical Laboratory, Children’s Hospital, Zhejiang University School of Medicine, National Clinical Research Center for Child Health 36697 , Hangzhou, China; Nationwide Children's Hospital, Columbus, Ohio, USA

**Keywords:** *Mycoplasma pneumoniae*, RPA, CRISPR/Cas13a, detection, mutation

## Abstract

*Mycoplasma pneumoniae* (MP) is an important pathogen associated with respiratory tract infections. Macrolide-resistant MP infections (which mainly contain the A2063G mutation in their 23S rRNA) contribute to severe cases, complications, and sequelae development. Therefore, the development of a sensitive, rapid, and accurate method for MP detection and its A2063G mutation is critical for early differential diagnosis and for guiding clinical medication. Herein, we establish a one-step, highly efficient, and specific fluorescence-based detection system for the MP nucleic acid using clustered regularly interspaced short palindromic repeats (CRISPR)/Cas13a combined with recombinase polymerase amplification (RPA) isothermal technology. A detection limit of 5 copies/μL within 40 min was achieved using this system. When applied to 57 clinical specimens, this method exhibited a sensitivity of 96.3% and a specificity of 100% compared with quantitative real-time polymerase chain reaction (PCR). We also developed a two-step detection system for macrolide-resistant mutations with a sensitivity of 10 copies/μL. The RPA-CRISPR/Cas13a system was further applied to detect 27 clinical specimens, and the results were consistent with the sequencing data. Additionally, the RPA-CRISPR/Cas13a detection system was integrated with lateral flow assay (LFA) technology to develop an LFA-RPA-CRISPR/Cas13a detection system. In conclusion, our RPA-CRISPR/Cas13a detection system enabled rapid, sensitive, and highly efficient detection of MP and its mutation. This new method provides an efficient tool for clinical and point-of-care testing (POCT) to facilitate early treatment and infection control.

**IMPORTANCE:**

A rapid, sensitive, and highly efficient nucleic acid detection system for *Mycoplasma pneumoniae* (MP) was developed in this study by integrating clustered regularly interspaced short palindromic repeats (CRISPR)/Cas13a with recombinase polymerase amplification (RPA) technology. A two-step assay based on this technology was then further developed to identify the A2063G mutation in the 23S rRNA gene of MP that was associated with macrolide resistance. The proposed system was then validated using clinical samples, and it demonstrated consistency with conventional diagnostic and sequencing results. Therefore, the RPA-CRISPR/Cas13a system enabled rapid, sensitive, and cost-effective detection of MP and its drug resistance mutation. This method is a robust tool for clinical and point-of-care testing (POCT) applications. These systems will facilitate early treatment and infection control of MP.

## INTRODUCTION

*Mycoplasma pneumoniae* (MP) is a common pathogen associated with respiratory tract infections. The human population is vulnerable to MP, and children are especially susceptible, experiencing symptomatic infections, such as tonsillitis, pharyngitis, sinusitis, bronchitis, and pneumonia. In severe cases, it may cause symptoms that include bronchial spasm, acute respiratory distress syndrome (ARDS), myocarditis, and meningitis (1, 2). MP is the primary pathogen found among hospitalized children with community-acquired pneumonia, and this creates a heavy burden on children’s health and family finances (3, 4).

MP infection generally peaks in autumn and winter, with significant outbreaks occurring at intervals of approximately 3 to 7 years, each lasting several months (5). Epidemic peaks of MP infections were documented across multiple European and Asian countries in early 2020 (6). A resurgence of MP-associated respiratory infections was reported in multiple countries at the end of 2023, especially with heightened transmission among pediatric populations (7, 8). Currently, there is no effective vaccine available for MP infection, and macrolide antibiotics remain the primary therapeutic option for MP pneumonia treatment in pediatric populations (9). In recent years, resistance to macrolide antibiotics is a critical factor that contributes to the emergence of refractory *Mycoplasma pneumoniae* pneumonia (10). The binding site of macrolide antibiotics is located in domain V of the 23S rRNA of MP. Nucleotide mutations in this region, particularly at the 2,063, 2,064, 2,067, and 2,617 positions, reduce the drug-ribosome interaction. Among these, the A2063G transition is the most frequent and is associated with high-level macrolide resistance (11, 12). To curb MP transmission, it is essential to continuously monitor its infection and mutational status to enhance preventive measures and avoid the occurrence of larger-scale outbreaks or the development of severe complications.

The laboratory diagnosis of MP commonly includes culture, serological detection, and nucleic acid testing. The culture method is time-consuming and costly. Serological detection can lead to false-positive or false-negative results due to its low sensitivity and specificity (13). Polymerase chain reaction (PCR) has high sensitivity and specificity and has become the new “gold standard” for laboratory detection (14). However, PCR application depends on specific equipment and a trained operator. Targeted high-throughput sequencing (targeted next-generation sequencing) technology can provide more precise detection, but expensive equipment and complex operation significantly restrict its clinical application. Recently, isothermal amplification technology and clustered regularly interspaced short palindromic repeats (CRISPR) gene editing technology have experienced rapid development in the field of pathogen diagnosis. These technologies can rapidly and efficiently amplify nucleic acids at a constant temperature with low requirements for equipment and operation, which makes them significantly advantageous for pathogen diagnosis, especially for point-of-care testing (POCT) (15).

The CRISPR/Cas system consists of CRISPR arrays and associated CRISPR-associated (Cas) proteins (16). In 2016, Alexandra demonstrated the trans-cleavage activity of CRISPR for the first time, showing that Cas13a activated the trans-cleavage activity after binding to the target RNA (17). Subsequent studies have confirmed similar activity in Cas12a and Cas12b (18, 19). Researchers have established a highly sensitive and specific nucleic acid detection platform based on the characteristics of the CRISPR/Cas enzymes. Zhang’s team developed the Specific High-sensitivity Enzymatic Reporter Unlocking (SHERLOCK) method, a CRISPR/Cas13a-based platform (20, 21). Doudna’s group established DNA Endonuclease Targeted CRISPR *Trans* Reporter (DETECTR) methods based on CRISPR/Cas12a and Cas12f (18, 22). Deng’s team established a PAM-dependent dsDNA Target-activated Cas12f1 *Trans* Reporter (PDTCTR) detection platform using the CRISPR/Cas12f1_ge4.1 system (23). In recent years, researchers have combined the CRISPR/Cas system with other technical methods to develop higher sensitivity and high-throughput platforms. These technologies have included microfluidic chips, digital isothermal amplification, electrochemical sensing, and lateral flow test strips (24–26).

Some researchers have developed detection methods for MP based on loop-mediated isothermal amplification (LAMP) combined with lateral flow immunoassays or electrochemical sensors (27, 28). Liu established a one-step method for MP detection by combining LAMP technology with CRISPR-Cas12a, thereby improving LAMP’s detection specificity (29). Zhou developed a method to detect MP nucleic acids based on a combination of CRISPR/Cas12b and recombinase polymerase amplification (RPA) technology that had high sensitivity and specificity (30). He J used enzymatic recombinase amplification combined with Cas12a to establish a fluorescence-based method that included a strip test for MP detection, and the method was rapid, highly sensitive, and highly specific (31). Although previous studies have established efficient and rapid platforms for detecting MP based on CRISPR/Cas12 and Cas14a, there have been no reports on methods for detecting MP and the single-nucleotide polymorphisms of drug-resistant mutation sites based on CRISPR/Cas13a. The objective of this study is to establish a detection system by combining RPA with CRISPR/Cas13a, along with fluorescence detection and lateral flow assay (LFA) technology. This technology will provide support for MP infection control and the treatment of severe cases.

## MATERIALS AND METHODS

### Reagents and equipment

The RPA amplification kit was purchased from TwistDx. The LwaCas13a enzyme was obtained from Shanghai Huicheng Biotechnology Co., Ltd. The T7 RNA polymerase, RNase inhibitor, HiScribe T7 High-Yield RNA Synthesis Kit, and Monarch RNA Purification Kit were supplied by New England Biolabs. The colloidal gold universal detection kit, Milenia GenLine HybriDetect, was ordered from Mileniabiotec. The Mycoplasma genomic DNA extraction kit, the QIAamp UCP Pathogen Mini Kit, was sourced from Qiagen. The DNA marker DL1000, plasmid mini extraction kit, DNA fragment purification kit, and Probe qPCR Mix were supplied by the Takara Bio (Dalian) Co., Ltd. The rNTP mixture and RNase-free water were purchased from Sangon Biotech (Shanghai) Co., Ltd. *Legionella pneumophila, Klebsiella pneumoniae, Mycobacterium tuberculosis, Streptococcus pneumoniae,* human adenovirus, influenza A virus, influenza B virus, respiratory syncytial virus, Epstein-Barr virus, and MP strains were supplied by Bona Biotech.

The Bio-Rad Gel imaging system, the Eppendorf ThermoStat C constant temperature incubator, the real-time fluorescence quantitative PCR instrument ABI7500, the Droplet digital PCR machine QX200, the ABI Veriti 96-well thermal cycler, and the Thermo NanoDrop 2000 spectrophotometer.

### RPA amplification primer screening

Based on the principles of RPA primer design, the RPA amplification primers for the P1 and 23S rRNA genes of MP were designed using the National Center for Biotechnology Information Primer-BLAST website and Oligo software. These primers were synthesized by the Genscript Biotech Corporation. Recombinant plasmids that harbored the P1 gene, wild-type 23S rRNA gene, and the A2563G mutant 23S rRNA gene were constructed by the Shanghai Sangon Biotech Co., Ltd. Following the manufacturer’s instructions for the RPA amplification kit, a 50-μL amplification reaction was prepared by combining the following components: 29.5 μL of rehydration buffer, 2.4 μL each of 10 μM forward and reverse primers, 2 μL of gradient-diluted plasmid DNA, and RNase-free water to adjust the total volume to 47.5 μL. The reaction was initiated by adding 2.5 μL of a 280 mM magnesium acetate activator. Amplification was performed at 39°C for 50 min in a constant-temperature incubator. The products were purified using a DNA fragment purification kit and subsequently analyzed using 1.5% agarose gel electrophoresis. All sequences are listed in Table S1.

### crRNA screening

We designed a series of crRNAs based on the RPA-amplified fragment sequence and the preference rules of the Cas13a enzyme. The complementary single-stranded DNA templates of crRNA that contained T7 promoter sequences were annealed to form a double-stranded DNA. The annealing reaction was performed in a 10-μL mixture that contained 1 μL each of a 100 μM DNA template, 1 μL of a 10× Taq buffer, and 7 μL of nuclease-free water. The mixture was denatured at 95°C for 10 min in a PCR thermocycler. This was followed by a gradual decrease to 25°C at a rate of 0.1°C per 8 s to facilitate proper hybridization. The annealed products served as templates to generate crRNA using the HiScribe T7 High-Yield RNA Synthesis Kit. The crRNA products were treated with DNase I for 30 min to remove the DNA templates. They were then purified using the Monarch RNA Purification Kit. The crRNAs were quantified, aliquoted, and stored at −80°C.

### RPA-CRISPR/Cas13a fluorescence detection

The optimized RPA-CRISPR/Cas13a detection system contained 20 μL of reaction volume. The RPA reaction solution that included 1.6 μL each of forward and reverse primers (10 μM), 7.3 μL of nuclease-free water, and 29.5 μL of a rehydration buffer was added to the RPA single pellet aliquot to resuspend the enzyme mixture powder. The CRISPR/Cas13a detection mixture consisted of 0.5 μL crRNA (486 nM), 1.0 μL of RNA alert (10 μM), 0.5 μL of RNase inhibitor crRNA (40 U/μL), 0.1 μL of T7 RNA polymerase (40 U/μL), 0.2 μL of rNTP (25 mM), 1.5 μL of Cas13a (60 ng/μL), and 4.2 μL of nuclease-free water prepared to a 8 μL volume. In the one-step protocol, the reaction mixture was prepared and aliquoted (18 μL), followed by the addition of 1 μL of template DNA and 1 μL of 240 mM magnesium acetate. The two-step approach involved aliquoting 10 μL of the RPA reaction mixture supplemented with 1 μL of the template DNA and 1 μL of 240 mM magnesium acetate. This was followed by 5 min of incubation at 39°C. Subsequently, 8 μL of the CRISPR/Cas13a reaction mixture was rapidly added. The reactions were performed at 39°C for 60 min in ABI 7500 with minute-interval fluorescence monitoring.

### Sensitivity and specificity of the RPA-CRISPR/Cas13a system

The MP P1 gene and 23sRNA plasmids were diluted in a 10-fold gradient to the power of 10^−9^. The MP strain was serially diluted 10-fold across three gradients. The RPA-CRISPR/Cas13a system was then applied to detect various plasmid DNA concentrations and strain templates. In addition, various plasmid concentrations of the strain were also evaluated using quantitative real-time PCR (qPCR). Digital PCR was employed to quantify the various plasmid dilutions and the strains. The drug-resistant mutation of MP detection limit was determined using the mutant and wild-type plasmid DNA mixed in different ratios (proportion of the mutant plasmids: 100%, 50%, 10%, 1%, 0.1%, 0.01%), and was then detected using the RPA-CRISPR/Cas13a system.

The whole-genome nucleic acid of nine common respiratory pathogens was tested using the RPA-CRISPR/Cas13a system to evaluate the specificity. These non-MP pathogens were *Legionella pneumophila, Klebsiella pneumoniae, Mycobacterium tuberculosis, Streptococcus pneumoniae,* human adenovirus, influenza A virus, influenza B virus, respiratory syncytial virus, and the Epstein-Barr virus.

### RPA-CRISPR/Cas13a-based lateral flow assay

**Fig 1:**
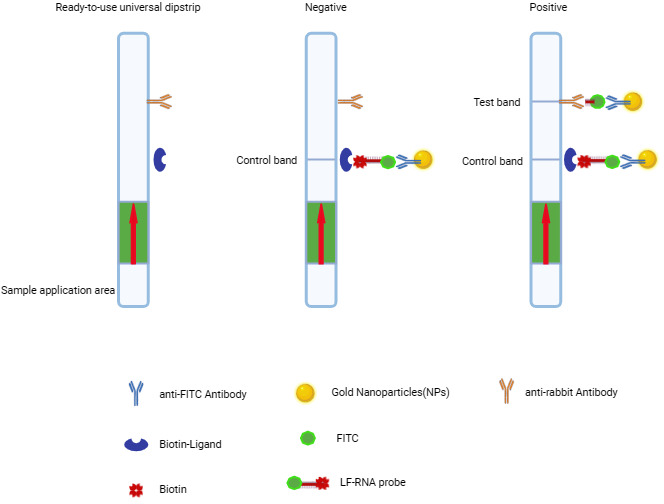
Principles of the lateral flow assay.

Nucleic acid lateral flow strips are an immunochromatographic technique that utilizes gold nanoparticles (AuNPs) to detect pathogen nucleic acids. In this system, the RPA-CRISPR/Cas13a reaction product is mixed with the analysis buffer of the Milenia HybriDetect kit, and the test strip is immersed for detection. The activated Cas13a enzyme cleaves the FAM- and biotin-labeled probes in the presence of the target sequence, thereby separating the FAM and biotin moieties. The cleaved FAM fragments in the analyte solution bind to the gold-conjugated anti-FAM antibodies in the sample application zone, forming a colloidal gold-antibody complex. The complex migrates along the membrane by capillary action. Intact FAM-biotin probes that are bound to the gold nanoparticles are captured by immobilized biotin ligands at the control zone (C), while the cleaved FAM-gold complexes are captured by species-specific antibodies at the test zone (T) (Fig. 1).

### Clinical sample application

A total of 57 human nasopharyngeal swab samples were collected from The Children’s Hospital Zhejiang University School of Medicine from February to March 2025, including 27 MP-positive samples and 30 non-MP samples (6 influenza A virus, 5 influenza B virus, 15 respiratory syncytial virus, and 4 adenovirus samples). These samples were detected using the RPA-CRISPR/Cas13a detection system to validate its performance. The 27 MP-positive samples were obtained from hospitalized pediatric patients presenting with symptomatic respiratory infections, alongside physical findings of moist rales and imaging evidence of pulmonary lesions. The 23S rRNA gene fragments of the MP-positive samples were amplified using PCR for sequencing, and the mutation sites were analyzed. These positive samples were then assayed utilizing the RPA-CRISPR/Cas13a-based A2063G detection system to validate its performance. The genomic DNA of the pathogen was extracted from the samples using the QIAamp UCP Pathogen Mini Kit according to the manufacturer’s instructions.

## RESULTS

### Establishment and optimization of the RPA-CRISPR/Cas13a system

**Fig 2:**
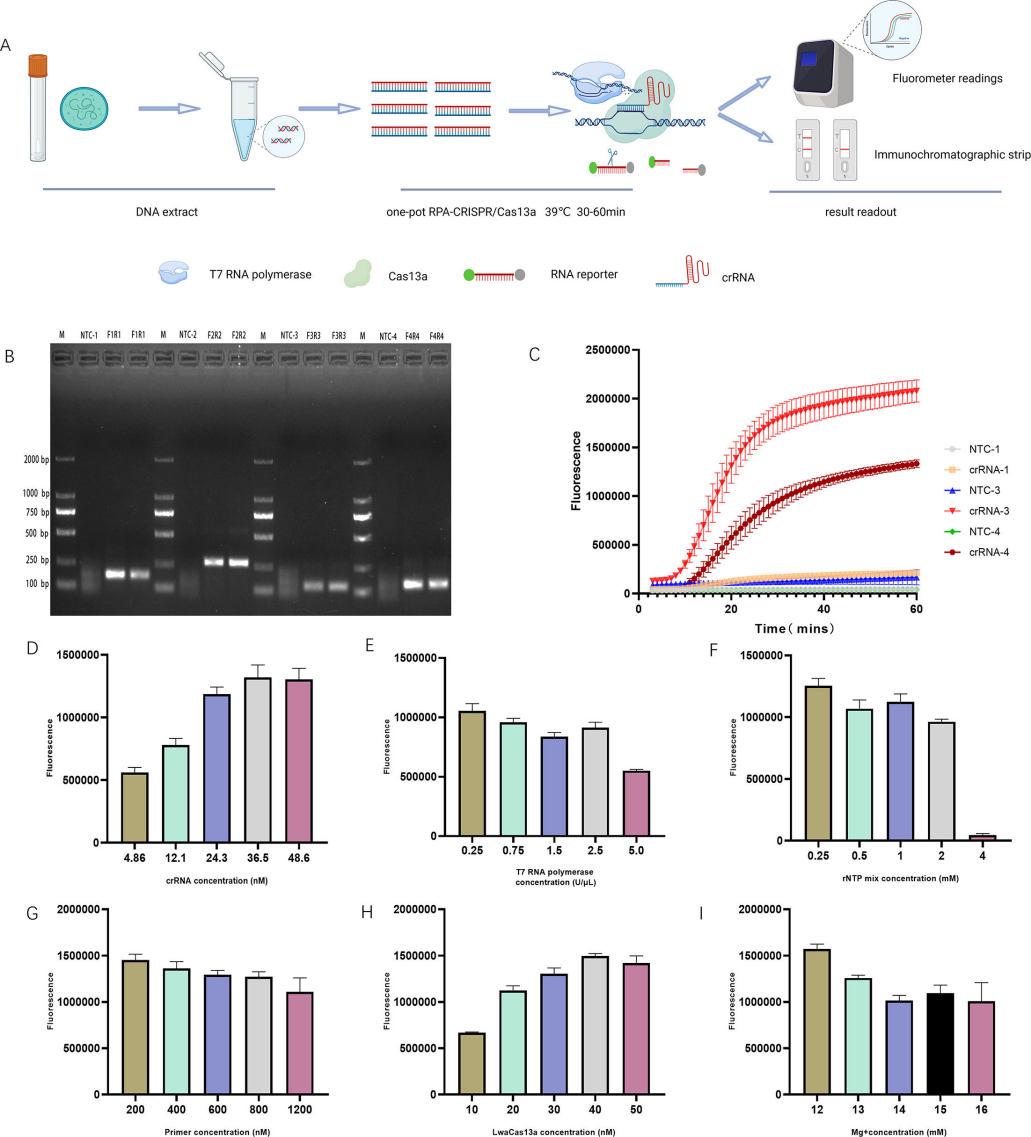
Optimization of the RPA-CRISPR/Cas13a testing. (**A**) Schematic workflow of the RPA-CRISPR/Cas13a-based nucleic acid detection for MP. (**B**) Agarose gel electrophoresis analysis for the screening of RPA amplification primers. M: DNA marker; F1R1−F4R4: replicate amplifications of the plasmid DNA template (diluted to 10^−6^). (**C**) Comparative evaluation of the RPA primers and matched CRISPR/Cas13a detection. Primer pair 3 combined with crRNA-3 demonstrated optimal performance for the detection of the plasmid template (diluted to 10^−7^). (**D–I**) The end-point fluorescence intensity of the RPA-CRISPR/Cas13a assay for optimization of critical components, including crRNA concentrations of 4.86, 12.1, 24.3, 36.5, and 48.6 nM; T7 RNA polymerase concentrations of 0.25, 0.75, 1.5, 2.5, and 5.0 U/μL; rNTP concentrations of 0.25, 0.5, 1.0, 2.0, and 4.0 mM; primer concentrations of 200, 400, 600, 800, and 1,200 nM; Cas13a enzyme concentrations of 10, 20, 30, 40, and 50 nM; and Mg^2+^ concentrations of 12, 13, 14, 15, and 16 mM. Average results from three biological replicates are shown. NTC, negative control; using nuclease-free water as a target DNA.

The RPA-CRISPR/Cas13a detection system primarily involves the incorporation of a T7 promoter sequence at the 5′ terminus of the RPA forward primer. The RPA products serve as a template for T7 RNA polymerase-mediated transcription to generate target RNA molecules. This RNA product is recognized and bound by the Cas13a-crRNA complex, leading to the activation of Cas13a. The activated Cas13a exhibits collateral cleavage activity, indiscriminately degrading fluorescence-labeled RNA reporter probes. The result can be quantitatively interpreted using fluorescence intensity measurements or assessed via visual inspection (Fig. 2A).

The P1 protein of MP is a membrane surface protein that possesses antigenicity and immunogenicity, and it plays a crucial role in cell adhesion and pathogenicity. We selected the conserved sequence of the P1 gene as the detection target and designed four pairs of RPA amplification primers. Gel electrophoresis analysis of the RPA amplification products showed efficient amplification of all four primer pairs (Fig. 2B). The amplicon sequence of primer pair 2 was longer; hence, we chose amplicons of primer pairs 1, 3, and 4 as templates to design the crRNA sequences and transcribed them *in vitro* to obtain the crRNA. We referred to Zhang Feng’s SHERLOCK detection system, and the crRNA was diluted to a working concentration of 10 ng/μL and incorporated into the RPA-CRISPR/Cas13a reaction system. The reaction was performed at 39°C for 60 min in the ABI7500 instrument. The results indicated that crRNA-3 exhibited optimal performance (Fig. 2C).

Key components of the reaction system were individually optimized by systematically adjusting the concentration of a single parameter at a time to enhance the detection efficiency. These included crRNA, T7 RNA polymerase, the rNTP mixture, primers, LwaCas13a, and the Mg^2+^ working concentrations (Fig. 2D through I). The cost-effective and optimal concentration for each component was confirmed. The results indicated that 36.5 nM crRNA, 0.25 U/μL T7 RNA polymerase, 0.25 mM rNTP mix, 200 nM primers, 40 nM LwaCas13a, and 12 mM Mg^2+^ generated the best performance.

### Sensitivity and specificity analysis for the RPA-CRISPR/Cas13a system

**Fig 3:**
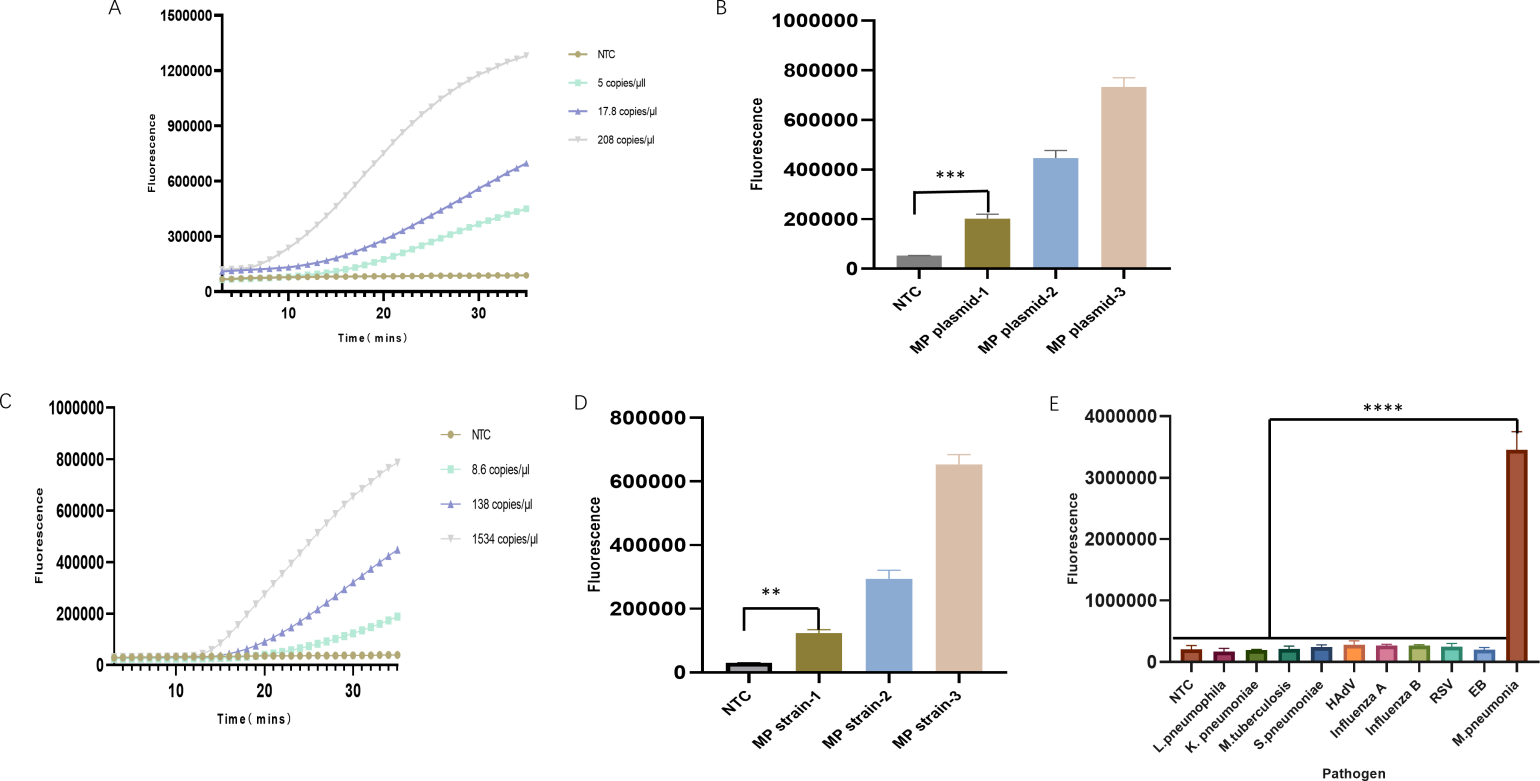
RPA-CRISPR/Cas13a sensitivity and specificity analyses for the detection of MP. (**A–D**) Sensitivity was assessed by testing three concentrations of the MP plasmid and MP strain. (**E**) Specificity was validated by testing nine non-MP pathogens. The non-MP pathogens were *Legionella pneumophila, Klebsiella pneumoniae, Mycobacterium tuberculosis, Streptococcus pneumoniae,* human adenovirus, influenza A virus, influenza B virus, respiratory syncytial virus, and the Epstein-Barr virus. The MP strain served as the positive control. The results represent the average of three biological replicates. **P < 0.01; ***P < 0.001; ****P < 0.0001.

The sensitivity and specificity of the RPA-CRISPR/Cas13a detection system were assessed after the system was optimized. The MP strain concentration was labeled as 10^4^–10^5^ copies/μL and was serially diluted 10-fold across three gradients. The P1 gene plasmid was performed in 10-fold gradient dilutions to the power of 10^−9^. The primer and probe for qPCR were evaluated by testing different dilution gradients of plasmids and MP strains. Digital PCR was used to quantify various dilutions of both the plasmid and strain (Table 1). The RPA-CRISPR/Cas13a system was then applied to detect the three lowest concentrations. The results showed a detection limit of 5 copies/μL for MP P1 plasmid and 8.6 copies/μL for MP strain using the RPA-CRISPR/Cas13a system (Fig. 3A through D).

**TABLE 1:** Concentrations of various dilutions of plasmids and MP strains^*a*^

Target	Dilution (fold)	Ct value	Concentration (copies/μL)
MP plasmid-1	10^−9^	37	5
MP plasmid-2	10^−8^	34	17.8
MP plasmid-3	10^−7^	29	208
MP strain-1	10^−4^	36	8.6
MP strain-2	10^−3^	33	138
MP strain-3	10^−2^	30	1,534

^*a*^ Digital PCR was used to quantify the MP-plasmid (10^−9^–10^−7^) and MP-strain (10^−4^–10^−2^).

The RPA-CRISPR/Cas13a system specificity was verified by testing multiple bacterial strains or viral isolates (10^3^–10^4^ copies/μL). The results showed that the system identified the MP strain without cross-reactivity with non-target pathogens, indicating its high specificity (Fig. 3E).

### Establishment of the RPA-CRISPR/Cas13a system for detecting the drug-resistant mutation of MP

**Fig 4:**
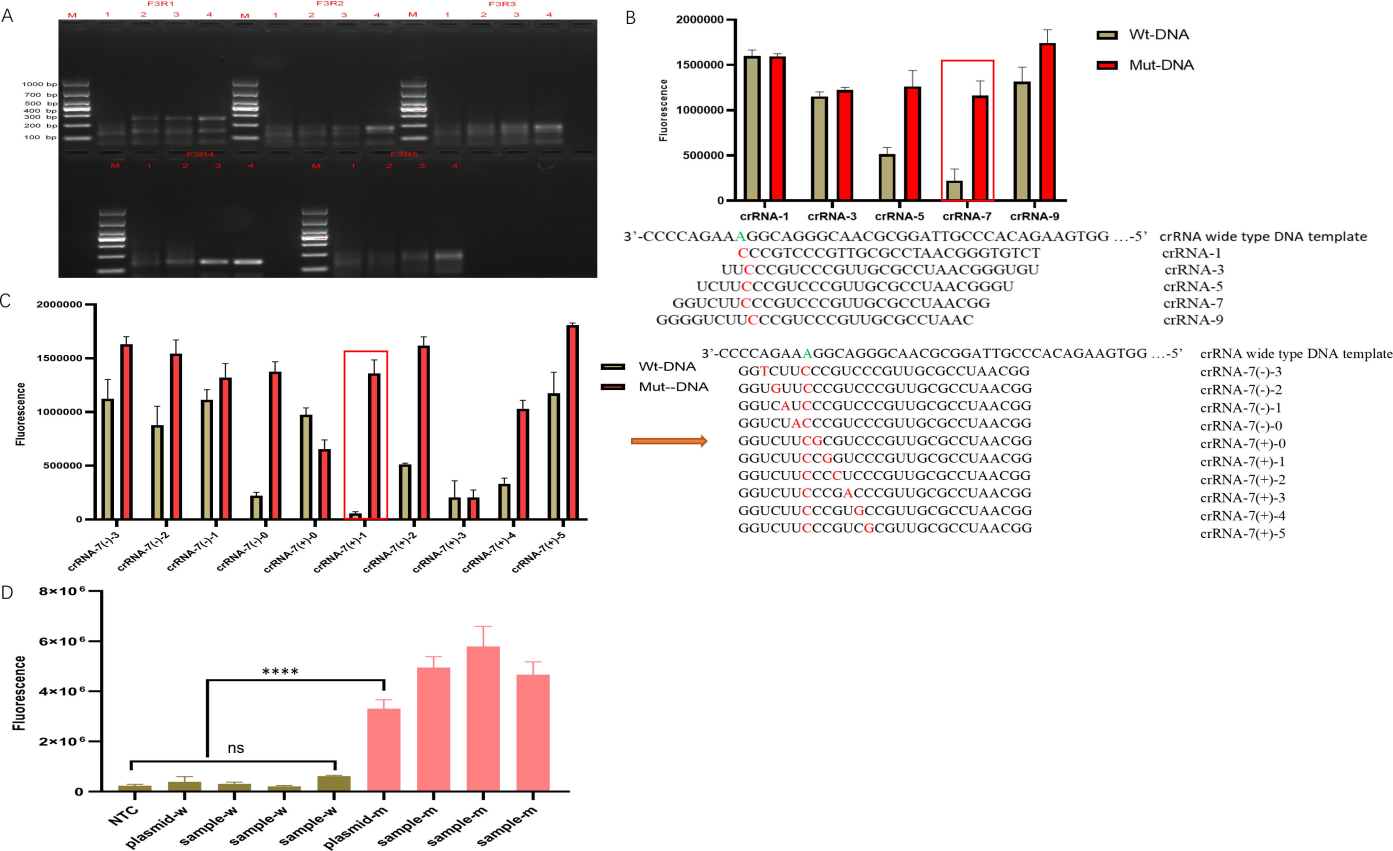
crRNA screening for MP drug-resistant mutations using the RPA-CRISPR/Cas13a. (**A**) RPA primer validation for the 23S rRNA A2063G mutation detection. Gel electropherogram shows the amplification products from five primer pairs (F3R1–F3R5) by using serially diluted 23S rRNA plasmid templates (10^−8^ to 10^−5^; lanes 1−4). M: DNA ladder. (**B**) First-round crRNA screening. The detection efficiency varied when the target mutation (A2063G) was positioned at different locations within the crRNA spacer. (**C**) Second-round crRNA screening. The detection specificity was further optimized by introducing additional mismatches flanking the target mutation. (**D**) Detection of selected crRNA in both wild-type and mutant MP samples. ns, *P* > 0.05; ****P < 0.0001.

The RPA primer sequences were designed within the genomic region that flanked the A2063G mutation site. RPA amplification screening was used to identify the optimal primer pair, 23s-F3R4, with obvious and special amplification (Fig. 4A).

Studies have demonstrated that Cas13a activity is influenced by the specific sequence and mutation type of the binding target. A single mismatch of some sequences may only lead to a partial decrease in activity, and continuous or multiple mismatches will significantly inhibit Cas13a activity (32). Based on this principle, we designed and screened crRNAs complementary to the 23S rRNA gene A2063G mutation site. In the first round of screening, the A2063G mutation site was introduced at the position of the first, third, fifth, seventh, and ninth nucleotides of the crRNA spacer. Fluorescence analysis in the RPA-CRISPR/Cas13a detection that used wild-type and mutant 23S rRNA plasmid templates revealed that the seventh-position mutation exhibited a superior detection performance (Fig. 4B). In the second round of optimization, the seventh-position mutation of the crRNA spacer was designated as the origin (position 0), with four upstream and six downstream nucleotides systematically altered to generate dual mismatches with the wild-type sequence. A fluorescence analysis during the RPA-CRISPR/Cas13a detection demonstrated that crRNA-7(+)-1 achieved the optimal detection efficacy (Fig. 4C). To compare the discriminatory performance of the selected crRNA-7(+)-1, we further detected both wild-type and mutant MP samples with the RPA-CRISPR/Cas13a system. The results showed that the crRNA could be used to significantly distinguish between the wild-type and mutant samples (Fig. 4D).

### RPA-CRISPR/Cas13a detection for the drug-resistant mutation sensitivity and specificity analyses

**Fig 5:**
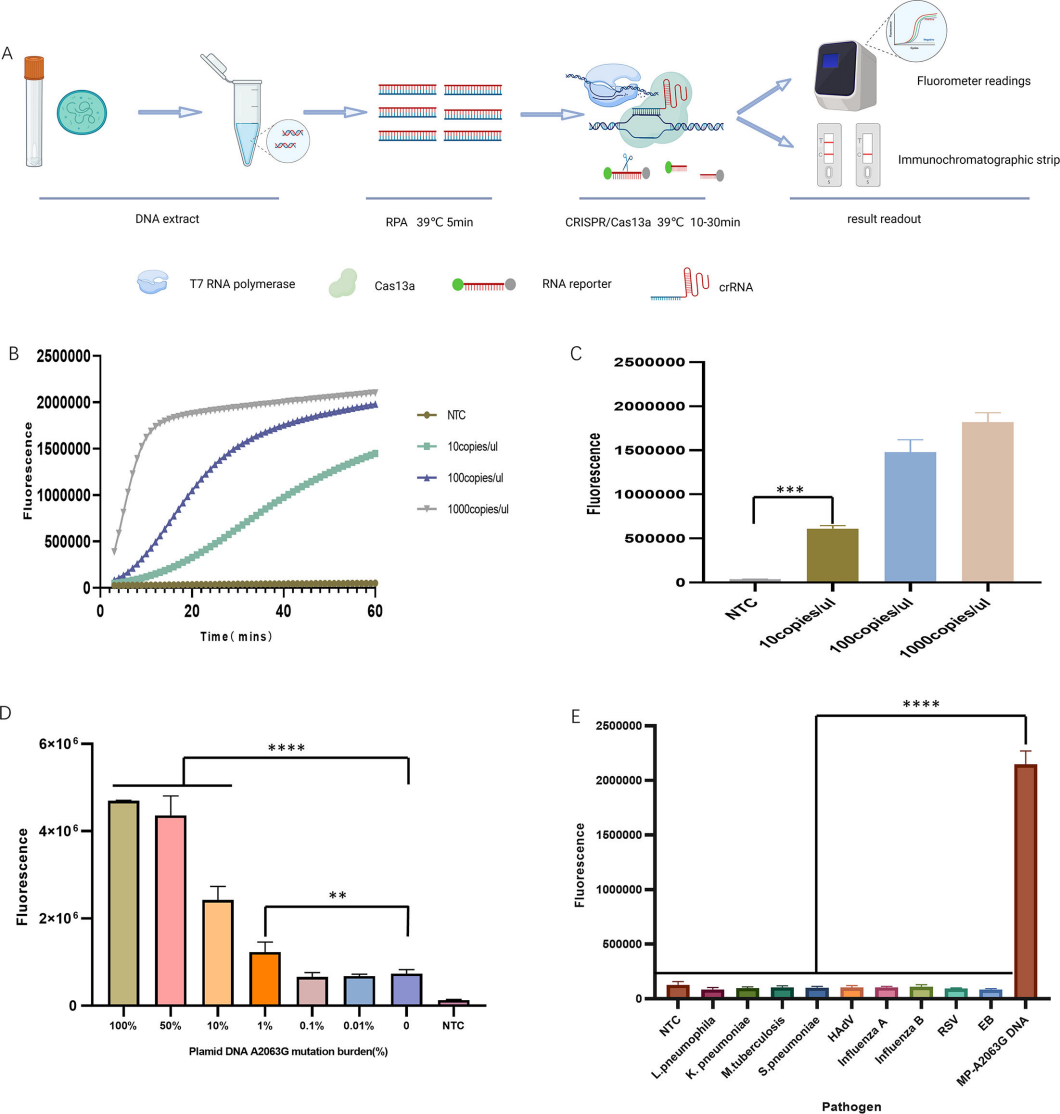
RPA-CRISPR/Cas13a for the detection of the drug resistance mutation, sensitivity, and specificity analyses. (**A**) Schematic workflow of the two-step RPA-CRISPR/Cas13a assay for the identification of the drug-resistance mutation. (**B and C**) Sensitivity analysis showing the fluorescence signals of RPA-CRISPR/Cas13a mutation detection for the serial dilutions of the template DNA (10, 100, and 1,000 copies per μL). (**D**) Detection of drug-resistant mutation rates with a sensitivity of 1%. (**E**) Specificity assessment demonstrating the fluorescence signals of the RPA-CRISPR/Cas13a nucleic acid mutation detection. **P < 0.01; ***P < 0.001; ****P < 0.0001.

We evaluated the sensitivity and specificity of the RPA-CRISPR/Cas13a system by targeting the A2063G mutation in the 23S rRNA gene. For the sensitivity testing, serial dilutions of the mutant 23S rRNA plasmid DNA (10–10^3^ copies/μL) were analyzed by using the two-step RPA-CRISPR/Cas13a assays (Fig. 5A). The two-step method demonstrated a superior sensitivity (10 copies/μL). The limit of detection for the A2063G mutation was also determined using the RPA-CRISPR/Cas13a system. The results showed that the detection system could achieve a sensitivity of 1%, which means it could detect 10 copies of the A2063G mutation among 1,000 copies of wild-type MP (Fig. 5B through D). Specificity was assessed against a panel of non-MP strains (10^6^–10^7^ copies/mL), with the mutant plasmid as a positive control. No cross-reactivity was observed, confirming its high specificity for detecting the A2063G mutation (Fig. 5E).

### MP clinical sample detection using the RPA-CRISPR/Cas13a system

We collected a total of 57 throat swab samples, including 27 MP-positive and 30 non-MP samples confirmed by PCR, to evaluate the analytical performance of the RPA-CRISPR/Cas13a system. The partial sequence of the 23S rRNA gene from the 27 MP-positive samples was amplified using PCR and analyzed via Sanger sequencing to characterize mutations in these samples (data not shown). The sequencing revealed 11 wild-type and 16 A2063G-mutant samples.

**Fig 6:**
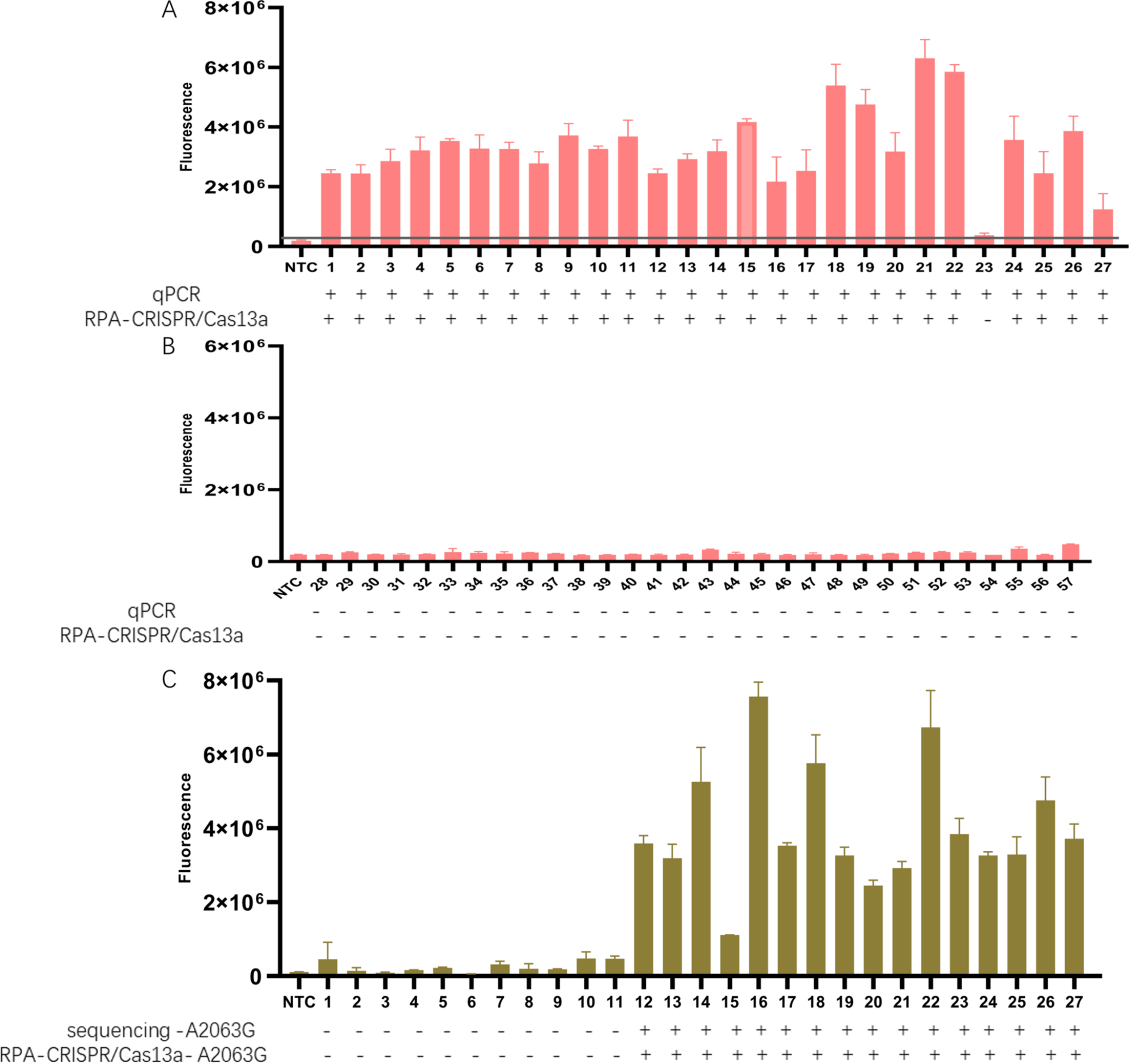
Application of the RPA-CRISPR/Cas13a system for clinical sample testing. (**A**) Fluorescence intensity histogram of 27 MP-positive clinical samples detected by the RPA-CRISPR/Cas13a detection system. (**B**) Fluorescence intensity histogram of 30 non-MP clinical samples detected by the RPA-CRISPR/Cas13a mutation detection system. (**C**) Fluorescence intensity histogram of 27 MP-positive clinical samples detected by the RPA-CRISPR/Cas13a mutation detection system.

The RPA-CRISPR/Cas13a system was applied to detect the 57 samples. The results demonstrated that the RPA-CRISPR/Cas13a detection identified 26 positive samples and 31 negative samples. For the mutation detection, the RPA-CRISPR/Cas13a system showed no amplification in the wild-type samples (1–11) but clear signals in the mutant samples (12–27) (Fig. 6). The results of clinical qPCR and sequencing data were taken as the control. Overall, the detection results of the RPA-CRISPR/Cas13a system were largely consistent with the controls. The RPA-CRISPR/Cas13a system exhibited a sensitivity of 96.3% and a specificity of 100% for diagnosis (Table 2).

**TABLE 2:** Sensitivity and specificity of the RPA-CRISPR/Cas13a system for the detection of MP^*a*^

		qPCR		
RPA-CRISPR/Cas13a system		Positive	Negative	Total
	Positive	26	0	26
	Negative	1	30	31
Total		27	31	

^*a*^ Sensitivity of the RPA-CRISPR/Cas13a system: 96.3% (95% CI: 81%–99.6%). Specificity of the RPA-CRISPR/Cas13a system: 100% (95% CI: 88.4%–100%). Positive predictive value of the RPA-CRISPR/Cas13a system: 100% (95% CI: 86.8%–99.5%). Negative predictive value of the RPA-CRISPR/Cas13a system: 96.8% (95% CI: 81.4%–99.6%). Diagnostic accuracy of the RPA-CRISPR/Cas13a system: 98.3% (95% CI: 90.7%–100%).

### Application of the RPA-CRISPR/Cas13a detection system combined with LFA in clinical samples

The established RPA-CRISPR/Cas13a detection systems utilized a fluorescence-based readout that still required specialized fluorometric equipment. To facilitate rapid, simple, and field-deployable detection of MP, we modified the systems by replacing the fluorescent RNA probe with an LFA-RNA Alert probe labeled with a 5′-FAM fluorophore and 3′-biotin, while retaining all other components, thereby establishing the LFA-RPA-CRISPR/Cas13a assay.

**Fig 7:**
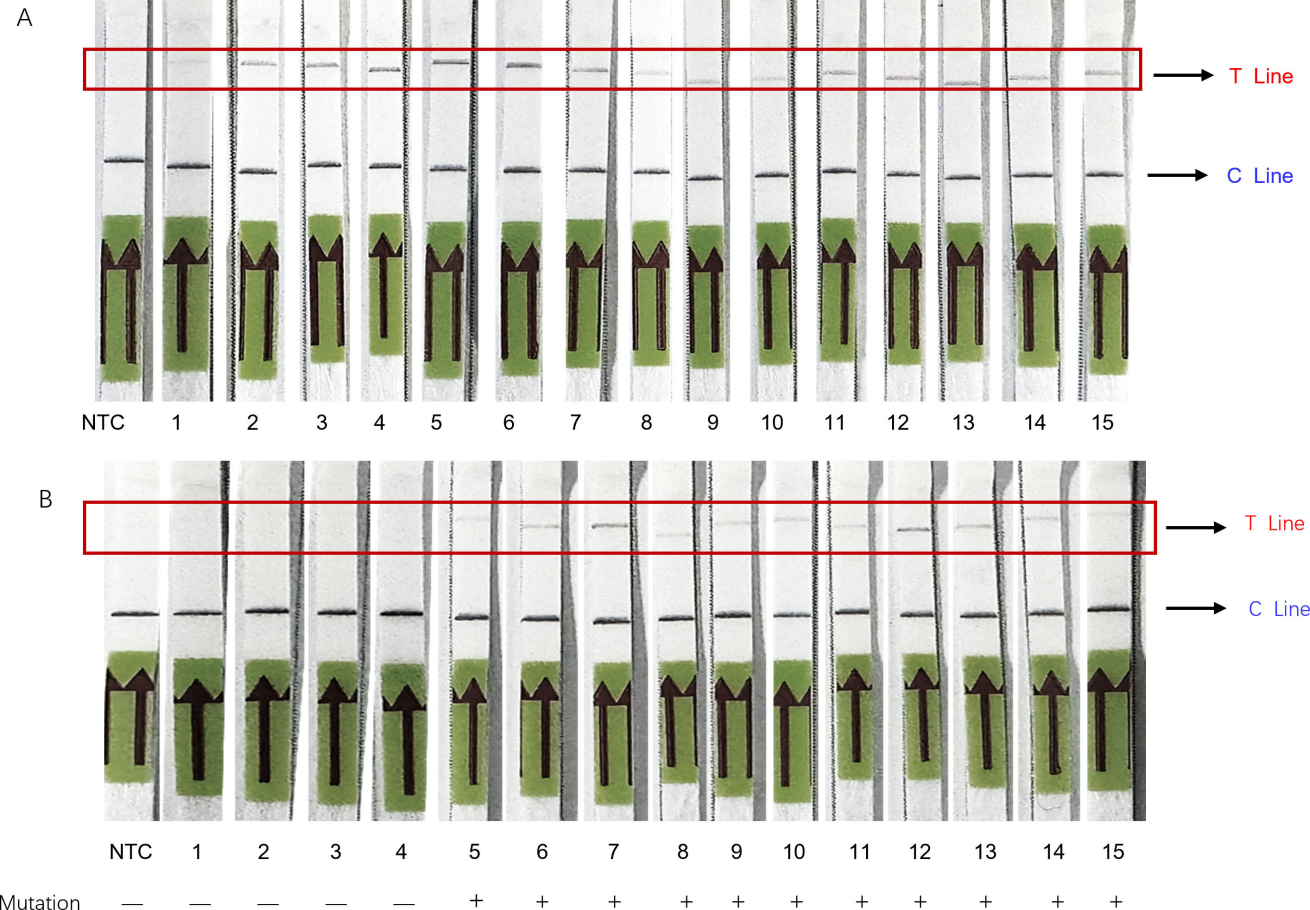
MP detection in clinical samples using the LFA-RPA-CRISPR/Cas13a assay. (**A**) LFA-RPA-CRISPR/Cas13a detection of 15 clinical samples. NTC, negative control; T, test line (positive signal); C, control line. (**B**) LFA-RPA-CRISPR/Cas13a-based mutation detection of 15 clinical samples. Samples 1–4 (wild type) showed no test line, while samples 5–15 exhibited T-bands of various intensities that correlated with the sequencing results.

For sample testing, 1 μL of genomic DNA was added to the RPA reaction mixture. This was followed by amplification at 39°C for 5 min. Subsequently, the LFA-CRISPR/Cas13a mixture was added to the RPA reaction solution and reacted at 39°C for 25 min. The product was then mixed with 80 μL of the Milenia GenLine HybriDetect assay buffer, incubated for 5 min at room temperature, and applied to a universal gold nanoparticle-based lateral flow strip. The results demonstrated that this system enabled accurate and rapid detection of the MP nucleic acids and the A2063G drug-resistant mutation (Fig. 7).

## DISCUSSION

MP is a common respiratory pathogen, particularly in children. Severe infections can lead to complications, such as bronchospasm, ARDS, myocarditis, and meningitis. Macrolide resistance is a critical factor in refractory and severe manifestations of MP infections. Studies have demonstrated that the A2063G mutation in the 23S rRNA gene serves as the primary genetic determinant of macrolide resistance. The primary methods for detecting macrolide-resistance mutation include qPCR and sequencing. However, qPCR has limitations in the identification of point mutations, while sequencing is time-consuming and costly. Currently, there are few studies regarding the application of CRISPR/Cas systems for MP mutation detection. Therefore, establishing a sensitive, rapid, and accurate method for detecting MP and its variants is essential for early differential diagnosis and effective treatment.

In this study, we established a one-step, rapid, and specific nucleic acid detection system for MP that integrates CRISPR/Cas13a with RPA isothermal amplification. Through systematic optimization of primers, crRNAs, and reaction conditions, the assay achieved a detection limit of 5 copies/µL. The specificity testing confirmed no cross-reactivity with non-target pathogens.

The Cas13a protein recognizes and cleaves complementary single-stranded RNA target sequences under the guidance of crRNA, thereby activating its collateral cleavage activity. The CRISPR/Cas13a enzyme does not require strict sequence preferences for its targeted cleavage sites, and when there are two or more mismatches between the crRNA and the target sequence, Cas13a is not activated. This characteristic provides CRISPR/Cas13a with greater flexibility and a broader target range for nucleic acid detection. We adapted this RPA-CRISPR/Cas13a system to detect the A2063G mutation of the 23S rRNA gene. After multiple screenings of the RPA primers and crRNA flanking of the mutation site, we compared the one-step and two-step approaches. There was a 10-fold sensitivity difference between one-step (100 copies/µL) and two-step (10 copies/µL) systems for the detection of the A2063G mutation. The established RPA-CRISPR/Cas13a detection systems were used to detect clinical samples, which were consistent with the qPCR and sequencing results. To enhance the field applicability, we combined the RPA-CRISPR/Cas13a system with LFA to establish an LFA-RPA-CRISPR/Cas13a platform that had good detection results.

The RPA-CRISPR/Cas13a detection platform demonstrated high sensitivity and specificity for the detection of MP and its mutation. We anticipate that it could aid in MP infection control and enable early treatment. However, its validation remains limited due to the relatively small number of clinical samples tested. Further verification and comparison with a larger sample are therefore necessary. The established A2063G mutation detection system requires a two-step assay that involves a tube opening, and this carries a potential risk of aerosol contamination. Consequently, there remains a need to integrate this system with other technologies to develop an integrated, closed-system approach, which can provide a highly efficient solution for both clinical and POCT applications.

## SUPPLEMENTAL MATERIAL

Table S1 spectrum.01793-25-s0001.xlsx The oligonucleotides used in this study.

ASM does not own the copyrights to Supplemental Material that may be linked to, or accessed through, an article. The authors have granted ASM a non-exclusive, world-wide license to publish the Supplemental Material files. Please contact the corresponding author directly for reuse.
